# Supplementary material for: A Critical View of the Application of the APEX Software (Aqueous Photochemistry of Environmentally-Occurring Xenobiotics) to Predict Photoreaction Kinetics in Surface Freshwaters
Source: Molecules. 2019 Dec 18;25(1):9. doi: 10.3390/molecules25010009 (PMC7017383; doi:10.3390/molecules25010009)
Supplement: Supplementary file 1 [file molecules-25-00009-s001.zip › 3.2.4_gcc-4.4.0.zip!/3.2.4_gcc-4.4.0/3.2.4_gcc-4.4.0/doc/octave/html/interpreter/Defining-Functions.html]

Defining Functions - Untitled


Next: Multiple Return Values,
Up: Functions and Scripts


---

### 11.1 Defining Functions

In its simplest form, the definition of a function named name
looks like this:

```
     function name
       body
     endfunction
```

A valid function name is like a valid variable name: a sequence of
letters, digits and underscores, not starting with a digit. Functions
share the same pool of names as variables.

The function body consists of Octave statements. It is the
most important part of the definition, because it says what the function
should actually *do*.

For example, here is a function that, when executed, will ring the bell
on your terminal (assuming that it is possible to do so):

```
     function wakeup
       printf ("\a");
     endfunction
```

The `printf` statement (see Input and Output) simply tells
Octave to print the string `"\a"`. The special character \a
stands for the alert character (ASCII 7). See Strings.

Once this function is defined, you can ask Octave to evaluate it by
typing the name of the function.

Normally, you will want to pass some information to the functions you
define. The syntax for passing parameters to a function in Octave is

```
     function name (arg-list)
       body
     endfunction
```

where arg-list is a comma-separated list of the function's
arguments. When the function is called, the argument names are used to
hold the argument values given in the call. The list of arguments may
be empty, in which case this form is equivalent to the one shown above.

To print a message along with ringing the bell, you might modify the
`wakeup` to look like this:

```
     function wakeup (message)
       printf ("\a%s\n", message);
     endfunction
```

Calling this function using a statement like this

```
     wakeup ("Rise and shine!");
```

will cause Octave to ring your terminal's bell and print the message
Rise and shine!, followed by a newline character (the \n
in the first argument to the `printf` statement).

In most cases, you will also want to get some information back from the
functions you define. Here is the syntax for writing a function that
returns a single value:

```
     function ret-var = name (arg-list)
       body
     endfunction
```

The symbol ret-var is the name of the variable that will hold the
value to be returned by the function. This variable must be defined
before the end of the function body in order for the function to return
a value.

Variables used in the body of a function are local to the
function. Variables named in arg-list and ret-var are also
local to the function. See Global Variables, for information about
how to access global variables inside a function.

For example, here is a function that computes the average of the
elements of a vector:

```
     function retval = avg (v)
       retval = sum (v) / length (v);
     endfunction
```

If we had written `avg` like this instead,

```
     function retval = avg (v)
       if (isvector (v))
         retval = sum (v) / length (v);
       endif
     endfunction
```

and then called the function with a matrix instead of a vector as the
argument, Octave would have printed an error message like this:

```
     error: value on right hand side of assignment is undefined
```

because the body of the `if` statement was never executed, and
`retval` was never defined. To prevent obscure errors like this,
it is a good idea to always make sure that the return variables will
always have values, and to produce meaningful error messages when
problems are encountered. For example, `avg` could have been
written like this:

```
     function retval = avg (v)
       retval = 0;
       if (isvector (v))
         retval = sum (v) / length (v);
       else
         error ("avg: expecting vector argument");
       endif
     endfunction
```

There is still one additional problem with this function. What if it is
called without an argument? Without additional error checking, Octave
will probably print an error message that won't really help you track
down the source of the error. To allow you to catch errors like this,
Octave provides each function with an automatic variable called
`nargin`. Each time a function is called, `nargin` is
automatically initialized to the number of arguments that have actually
been passed to the function. For example, we might rewrite the
`avg` function like this:

```
     function retval = avg (v)
       retval = 0;
       if (nargin != 1)
         usage ("avg (vector)");
       endif
       if (isvector (v))
         retval = sum (v) / length (v);
       else
         error ("avg: expecting vector argument");
       endif
     endfunction
```

Although Octave does not automatically report an error if you call a
function with more arguments than expected, doing so probably indicates
that something is wrong. Octave also does not automatically report an
error if a function is called with too few arguments, but any attempt to
use a variable that has not been given a value will result in an error.
To avoid such problems and to provide useful messages, we check for both
possibilities and issue our own error message.

— Built-in Function: **nargin** ()  
— Built-in Function: **nargin** (fcn\_name)  
> Within a function, return the number of arguments passed to the function.
> At the top level, return the number of command line arguments passed to
> Octave. If called with the optional argument fcn\_name, return the
> maximum number of arguments the named function can accept, or -1 if the
> function accepts a variable number of arguments.
>
>
>
> **See also:** nargout, varargin, varargout.

— Function File: **inputname** (n)  
> Return the text defining n-th input to the function.

— Built-in Function: val = **silent\_functions** ()  
— Built-in Function: old\_val = **silent\_functions** (new\_val)  
> Query or set the internal variable that controls whether internal
> output from a function is suppressed. If this option is disabled,
> Octave will display the results produced by evaluating expressions
> within a function body that are not terminated with a semicolon.
